# Orbital-scale denitrification changes in the Eastern Arabian Sea during the last 800 kyrs

**DOI:** 10.1038/s41598-018-25415-7

**Published:** 2018-05-04

**Authors:** Ji-Eun Kim, Boo-Keun Khim, Minoru Ikehara, Jongmin Lee

**Affiliations:** 1 Department of Oceanography, Pusan National University, Busan, 46241 Korea; 2 Center for Advanced Marine Research, Kochi University, Nankoku, 783-8502 Japan

## Abstract

Denitrification in the Arabian Sea is closely related to the monsoon-induced upwelling and subsequent phytoplankton production in the surface water. The δ^15^N values of bulk sediments collected at Site U1456 of the International Ocean Discovery Program (IODP) Expedition 355 reveal the orbital-scale denitrification history in response to the Indian Monsoon. Age reconstruction based on the correlation of planktonic foraminifera (*Globigerinoides ruber*) δ^18^O values with the LR04 stack together with the shipboard biostratigraphic and paleomagnetic data assigns the study interval to be 1.2 Ma. Comparison of δ^15^N values during the last 800 kyrs between Site U1456 (Eastern Arabian Sea) and Site 722B (Western Arabian Sea) showed that δ^15^N values were high during interglacial periods, indicating intensified denitrification, while the opposite was observed during glacial periods. Taking 6‰ as the empirical threshold of denitrification, the Eastern Arabian Sea has experienced a persistent oxygen minimum zone (OMZ) to maintain strong denitrification whereas the Western Arabian Sea has undergone OMZ breakdown during some glacial periods. The results of this study also suggests that five principal oceanographic conditions were changed in response to the Indian Monsoon following the interglacial and glacial cycles, which controls the degree of denitrification in the Arabian Sea.

## Introduction

Denitrification occurs when nitrate is used as an alternate electron acceptor to break down organic matter when dissolved oxygen concentration is lower than 0.2 ml/L^1^. During the denitrification, N_2_O and N_2_ are released into the atmosphere^2,3^. Thus, denitrification in the ocean plays an important role in the marine nitrogen cycle and global climate because it produces a greenhouse gas (N_2_O)^4^. OMZ develops in several oceans including the Eastern Tropical North Pacific, Eastern Tropical South Pacific, and Arabian Sea^5,6^. The OMZ in the Arabian Sea occurs between 200 to 1000 m in depth and accounts for more than 30% of the global denitrification process^2,6^. Denitrification in the Arabian Sea is generally associated with the monsoon climate, which leads to a seasonal reversal of wind pattern^6–9^. In the Western Arabian Sea, denitrification intensifies due to the enhanced primary production caused by strong upwelling which was induced by the southwest monsoon during summer^7,10^. During denitrification, lighter ^14^N-nitrate is utilized preferentially for the degradation of organic matter in the water column by bacteria, resulting in the increase of seawater nitrate δ^15^N values^11^. The controlling factors for δ^15^N value of marine sediments are diverse such as incomplete nitrate consumption^12^, Rayleigh isotopic fractionation^13^, early diagenesis^14^, and water column denitrification^15^. In general, the average δ^15^N value of global deep water is ~4.8‰ and increases with the degree of denitrification^16^. As a result, the δ^15^N values of seawater nitrate are higher than the average of global deep water when the degree of denitrification is strong in the Arabian Sea^17^. This change is reflected in the sediments below the water column by sinking of δ^15^N of sediment organic matter^8,18^. Previous studies reported that the empirical approach assigned ~6‰ as a reference or threshold δ^15^N value of sediment organic matter to judge the occurrence of denitrification^8,19,20^.

It is has been well known that the degree of denitrification fluctuates in response to the monsoon activity at seasonal-, millennial-, and orbital-scales in the Arabian Sea^6,7,9,10,20–25^ (Supplementary Figure S1). The Indian Monsoon system is one of the most distinctive features in the Arabian Sea. The southwest monsoon during summer develops by the low pressure in Peninsula India delivers rain^26^ and induces upwelling along the Oman Margin^27^. As a result, primary productivity increases^28^, seasonal development of the OMZ occurs^7^, and denitrification intensifies^7^. In contrast, the northeast monsoon prevails during winter^26^, causing upwelling along the west margin of India in a narrow portion of the Eastern Arabian Sea^29^. Thus, the δ^15^N value of sediment organic matter in the Arabian Sea increased during the interstadial and interglacial periods when the summer monsoon was enhanced to cause denitrification and the expansion of OMZ. In contrast, denitrification was relatively weakened and the OMZ was reduced during the stadial and glacial periods, when the winter monsoon was strong, resulting in a low δ^15^N value of sediment organic matter near ~4‰. However, previous studies in the Arabian Sea that revealed the relationship between the δ^15^N value of sediment organic matter and the degree of denitrification were limited spatially to shallow marine areas and temporally to millennial-scales^6,17,20,22,23,25^. In 2015, IODP Expedition 355 drilled the Laxmi Basin in the Eastern Arabian Sea in order to obtain long-term paleoceanographic records related to development of the Arabian Sea monsoon (i.e., Indian Monsoon)^30^. This study is the first to report the orbital-scale denitrification change associated with the monsoon activity throughout the Mid-Pleistocene in the Eastern Arabian Sea and discusses their differences between the Western and Eastern Arabian Seas in terms of diverse oceanographic factors that control the degree of denitrification.

## Study Area

**Figure 1:**
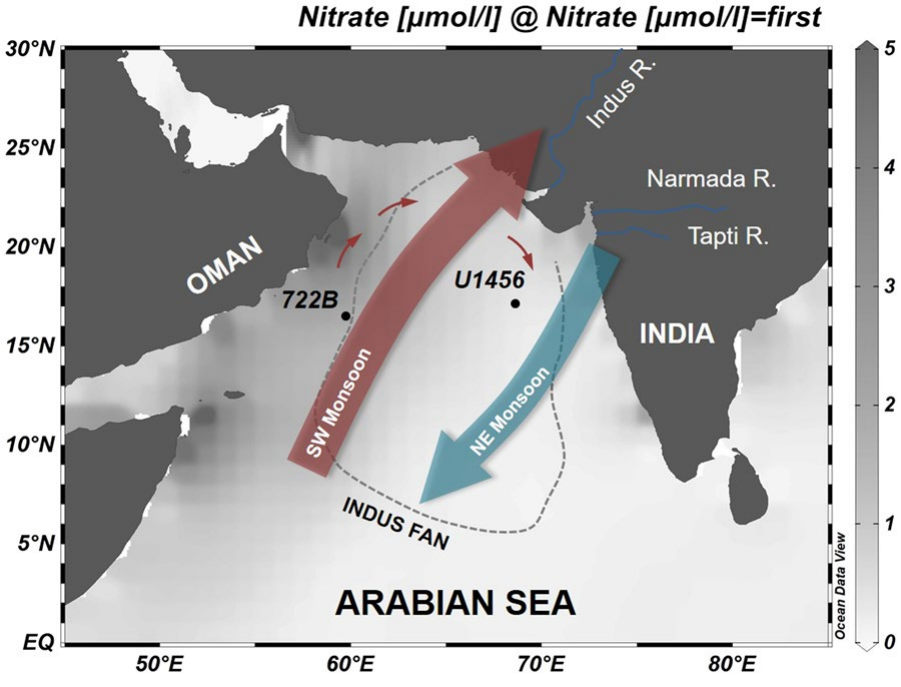
IODP Expedition 355 Site U1456 and ODP Leg 117 Site 722B in the Arabian Sea with seasonal wind patterns of the Indian Monsoon. Site U1456 (16°37.28′N, 68°50.33′E, 3640 m below sea level) is located in the Eastern Arabian Sea and Site 722B (16°37.31′N, 59°47.76′E, 2028 m below sea level) is situated in the Western Arabian Sea. The gradation in the sea represents annual nitrate content of the surface water in the Arabian Sea. The grey dotted line represents the outline of the Indus Fan. The small red arrow symbolizes the Somali Current in the far north of the Arabian Sea that flows clockwise when the southwest monsoon is strong.

IODP Expedition 355 Site U1456 is located in the Laxmi Basin of the Eastern Arabian Sea, within the mid-fan of the Indus Fan, which is the second largest fan system in the world (Fig. 1). The Indus Fan delta system is characterized by the repetition of turbidites that were formed by sediments supplied from various rivers adjacent to the Indian continent^31^. The Indus, Narmada and Tapti rivers in western India discharge more than 15,000 m^3^/s of freshwater into the Eastern Arabian Sea annually^32^. The warm-saline oxygen-depleted Red Sea (Persian Gulf) Intermediate Water (RSIW or PGIW) flows into the intermediate depth of the Arabian Sea^33,34^. The oxygen-rich Antarctic Intermediate Water (AAIW) also flows into the intermediate depth of the Arabian Sea from the south^34^.The intrusion of AAIW into the Arabian Sea is inhibited by lateral development of low-salinity and oxygen-rich Banda Sea Intermediate Water during the warm or interglacial periods, and *vice versa* during cold or glacial periods^35^.

## Results

**Figure 2:**
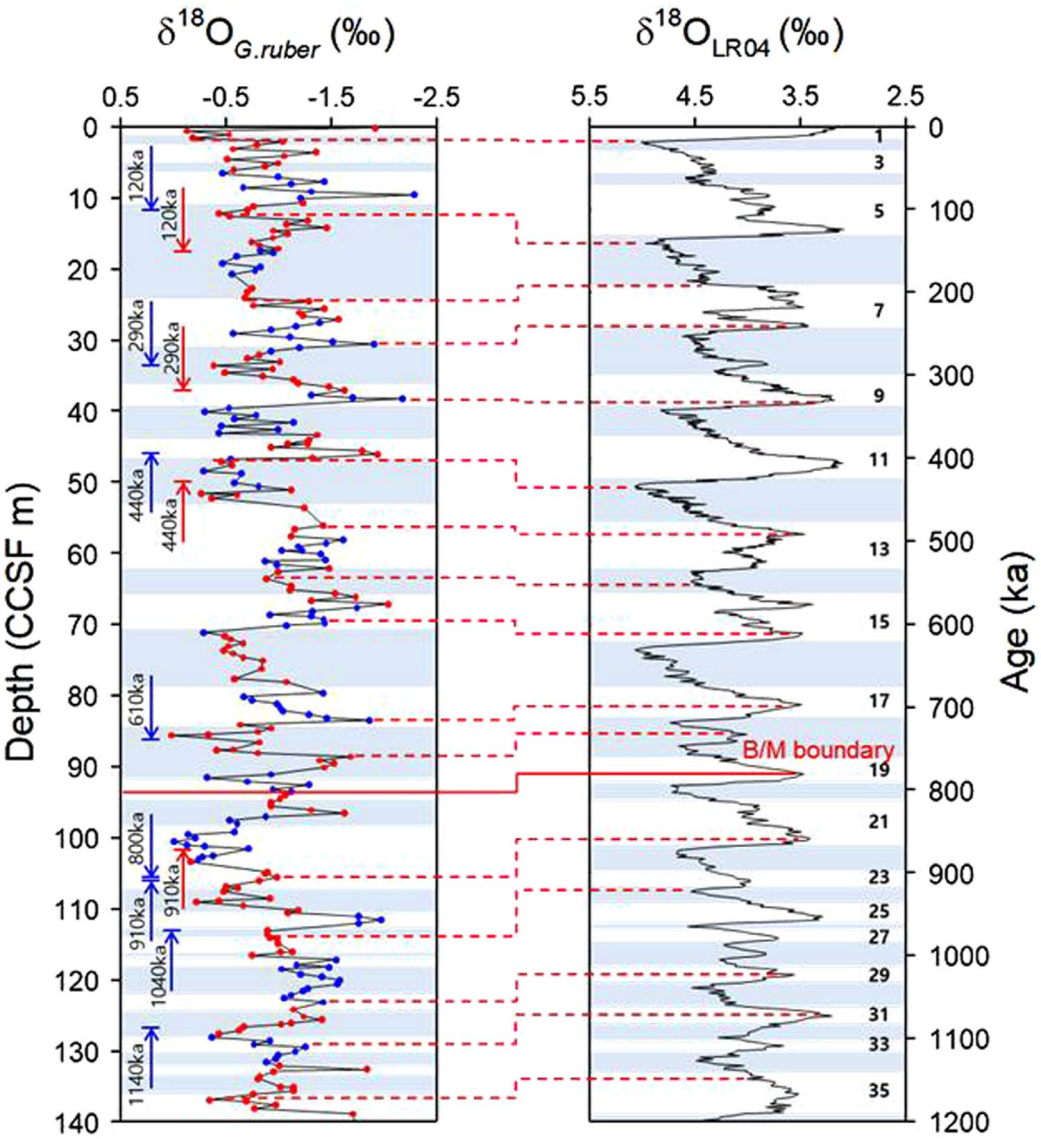
Age model of uppermost Unit I at Site U1456 reconstructed by δ^18^O values of planktonic foraminifera to correlate to LR04 stacks. The left figure depicts downcore variations in δ^18^O values of planktonic foraminifera (*G. ruber*) measured from Unit I at Site U1456 and the right figure shows LR04 stacks of benthic foraminiferal δ^18^O values^36^. The blue and red dots in the left figure are δ^18^O data from Holes U1456A and U1456C, respectively. The blue (Hole U1456A) and red (Hole U1456C) arrows represent the range of shipboard biostratigraphic datum^30^. The red solid line indicates the Brunhes-Matuyama (B/M) boundary (781 ka)^30^. The white interval represents interglacial periods characterized by low δ^18^O values, whereas the blue interval covers the glacial periods showing the high δ^18^O value. Detailed age reconstruction is explained in Supplementary Figure S3.

The sedimentary sequence drilled at Site U1456 was divided onboard into four lithologic units based on visual descriptions, magnetic susceptibility and colour spectral analysis^30^. The uppermost Unit I consisted mostly of hemipelagic to pelagic ooze with occasional turbidites composed of sand, silt, clay, and mixtures (Supplementary Figure S2). These turbidite layers are characterized by poor preservation of planktonic foraminifera and erosional contact of sediments, which may be obstacles to construction of a precise age model. Under such circumstances, the age of Unit I was mainly determined by the correlation of δ^18^O values of planktonic foraminifera (*Globigerinoides ruber*) with the LR04 stack^36^ supplement to the consideration of shipboard biostratigraphic and paleomagnetic data^30^ (Fig. 2). The 37 tie points are determined by correlating δ^18^O values to the LR04 stack^36^, with an additional correlation of δ^15^N values to ODP Leg 117 Site 722B^10^ in the Western Arabian Sea (Supplementary Figure S3). Although the age reference of Site 722B is different from the LR04 stack^37^, the minor offset between them seems insignificant. The precise age determination is described in detail in Supplementary Figure S3. As a result, the age of Unit I spans as old as 1.2 Ma, covering up to Marine Isotope Stage (MIS) 36 (Fig. 2). The oxygen isotope stratigraphy of Unit I shows that the quasi-cyclic pattern is characterized by 100-ka periodicity (Supplementary Figure S4), which is prominently more visible since the Mid-Pleistocene Transition (MPT). Thus, this study focuses particularly on the last 800 kyrs during which the glacial-interglacial cycles are distinctly discernible.

**Figure 3:**
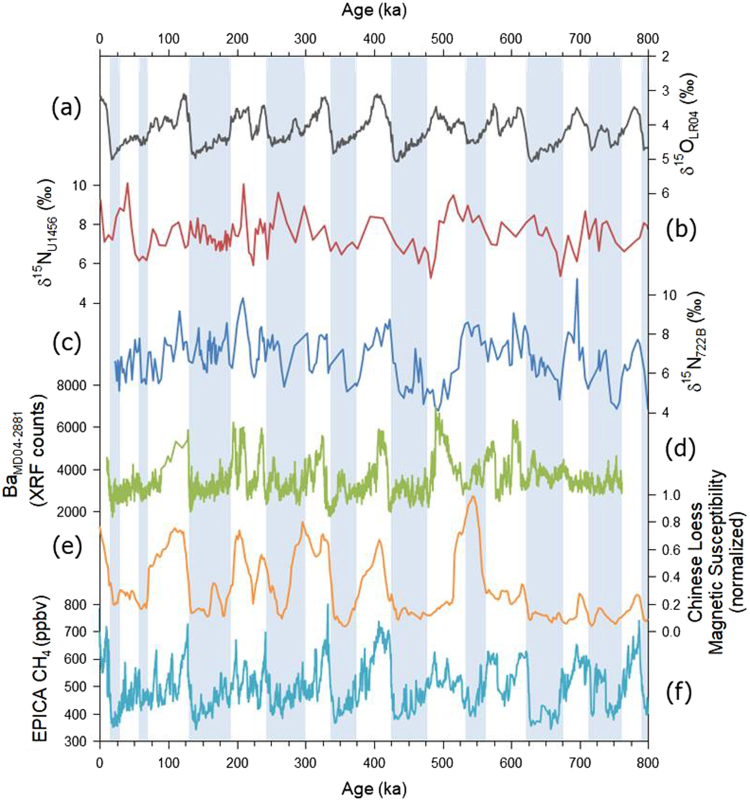
Orbital-scale variations in paleoclimate signals from various sites during the last 800 kyrs. (**a**) The interglacial-glacial periods defined by LR04 stacks of benthic foraminiferal δ^18^O values^36^. (**b**,**c**) Variation of δ^15^N values of bulk sediments at Sites U1456 (Eastern Arabian Sea) and 722B^10^ (Western Arabian Sea). (**d**) Barium content by XRF counting in core MD04-2881 in the Northeastern Arabian Sea, which represents the degree of primary productivity^25^. (**e**) Magnetic susceptibility of the Chinese loess plateau indicating the strength of the East Asian Monsoon^45^. (**f**) CH_4_ concentrations of the EPICA Dome C that are related to inland precipitation^47,48^.

The δ^15^N values of bulk sediments at Site U1456, representing the degree of denitrification in the Eastern Arabian Sea, range from 5.3 to 10.1‰ with an average of 7.5‰ during the last 800 kyrs (Fig. 3). The δ^15^N values at Site 722B in the Western Arabian Sea fluctuate similarly between 4.1 and 10.8‰ with an average of 6.6‰^10^ (Fig. 3). The δ^15^N variation between Site U1456 and Site 722B is comparable following glacial-interglacial changes, being characterized by high δ^15^N values during the interglacial periods indicating active denitrification and showing the opposite during glacial periods. Such data of δ^15^N from Site U1456 shows the 100-ka periodicity that is displayed in glacial-interglacial variations that are possible to be correlated to the orbital-scale eccentricity cycle. Such cyclicity is evident especially before the MPT for both δ^18^O and δ^15^N.

## Discussion

Tripathi *et al*.^19^ referred to 6‰ as indicative of the initiation of denitrification during the Pliocene and Miocene at Site U1456 in the Eastern Arabian Sea. Based on our results, the δ^15^N values at Site U1456 rarely fall below 6‰ (Fig. 3), which clearly indicates that denitrification occurred continuously during the last 800 kyrs in the Eastern Arabian Sea. On the contrary, the δ^15^N values at Site 722B are less than 6‰ during sporadic intervals, corresponding to the glacial periods. These findings indicate that denitrification has collapsed frequently during cold periods in the Western Arabian Sea^10^.

Nitrate utilization in the surface water affects the resultant δ^15^N value of sediment organic matter^11,16^. The nitrate concentration of the surface water between the Western and Eastern Arabian Sea offsets ~2 µM^11,38^, reflecting almost complete nitrate utilization during the phytoplankton bloom. The offset may increase seasonally and during the production, suggesting that variability of δ^15^N values would be predominantly driven by changes in nitrate consumption and not the initial nitrate δ^15^N value (partially set by water column denitrification). Thus, the δ^15^N values of sediment organic matter were influenced by the extent of denitrification within the OMZ, although nitrate utilization during the glacial periods was assumed to be similar to the present-day utilization. Hence, we accept the δ^15^N values of bulk sediments to compare the degree of denitrification between the Western (Site 722B) and Eastern (Site U1456) Arabian Sea. Because early diagenesis causes an increase of δ^15^N value during the degradation of sediment organic matter^39^, the possible effects of early diagenesis need to be evaluated to confirm that the alteration of δ^15^N values was caused solely by water column denitrification^14^. For example, Pichevin *et al*.^20^ reported that the δ^15^N value of sediment organic matter was affected by the depth of sedimentation and the degree of sediment organic matter degradation, both of which are related to early diagenesis. At Site U1456, the effect of early diagenesis was estimated by comparing the δ^15^N value with the total nitrogen (TN) content^40^ (Supplementary Figure S5). The lack of correlation between two parameters demonstrates that early diagenesis has not been a significant factor to raise δ^15^N values. Hence, the δ^15^N values of bulk sediments at Site U1456 are related to the degree of denitrification and development of OMZ within the water column.

Denitrification in the Eastern Arabian Sea during the last 800 kyrs has clearly varied between glacial and interglacial periods, which is closely related to the orbital-scale global climate changes (Fig. 3). Hence, we compared several monsoon-related proxies to evaluate the correspondence between denitrification and monsoon intensity. The degree of denitrification at Site 722B in the Western Arabian Sea has also changed comparably to that in the Eastern Arabian Sea. XRF-scanned Ba counts from core MD04-2881 collected in the Northeastern Arabian Sea increased during warm interglacial periods because the southwest monsoon drove the intense mixture between the surface and subsurface waters, resulting in enhanced primary productivity^25^. The increased production of organic matter promotes denitrification via more consumption of dissolved oxygen during degradation process. This relationship between surface water productivity and denitrification has also been recognized in the Eastern Tropical North Pacific and the Eastern Tropical South Pacific^5,6,41–44^. The Chinese loess plateau in south central China records glacial-interglacial fluctuations of the East Asian Monsoon which is closely linked to the Indian Monsoon^45^. Magnetic susceptibility (MS) during arid and cold glacial periods was low due to increased wind-blown loess, whereas MS during wet and warm interglacial periods was high because of soil-producing weathering^46^. Methane concentrations measured from EPICA Dome C (Antarctica) are closely linked to monsoon precipitation^47,48^. The strong global monsoon-induced precipitation expanded swamps and wetland in continents to release more methane into the atmosphere. Thus, global paleoclimate proxies related to the southwest monsoon were maximized during warm interglacial periods in the Arabian Sea, while those related to the northeast monsoon were minimized during cold glacial periods, which supports our finding that δ^15^N values of bulk sediments indicate the degree of denitrification.

**Figure 4:**
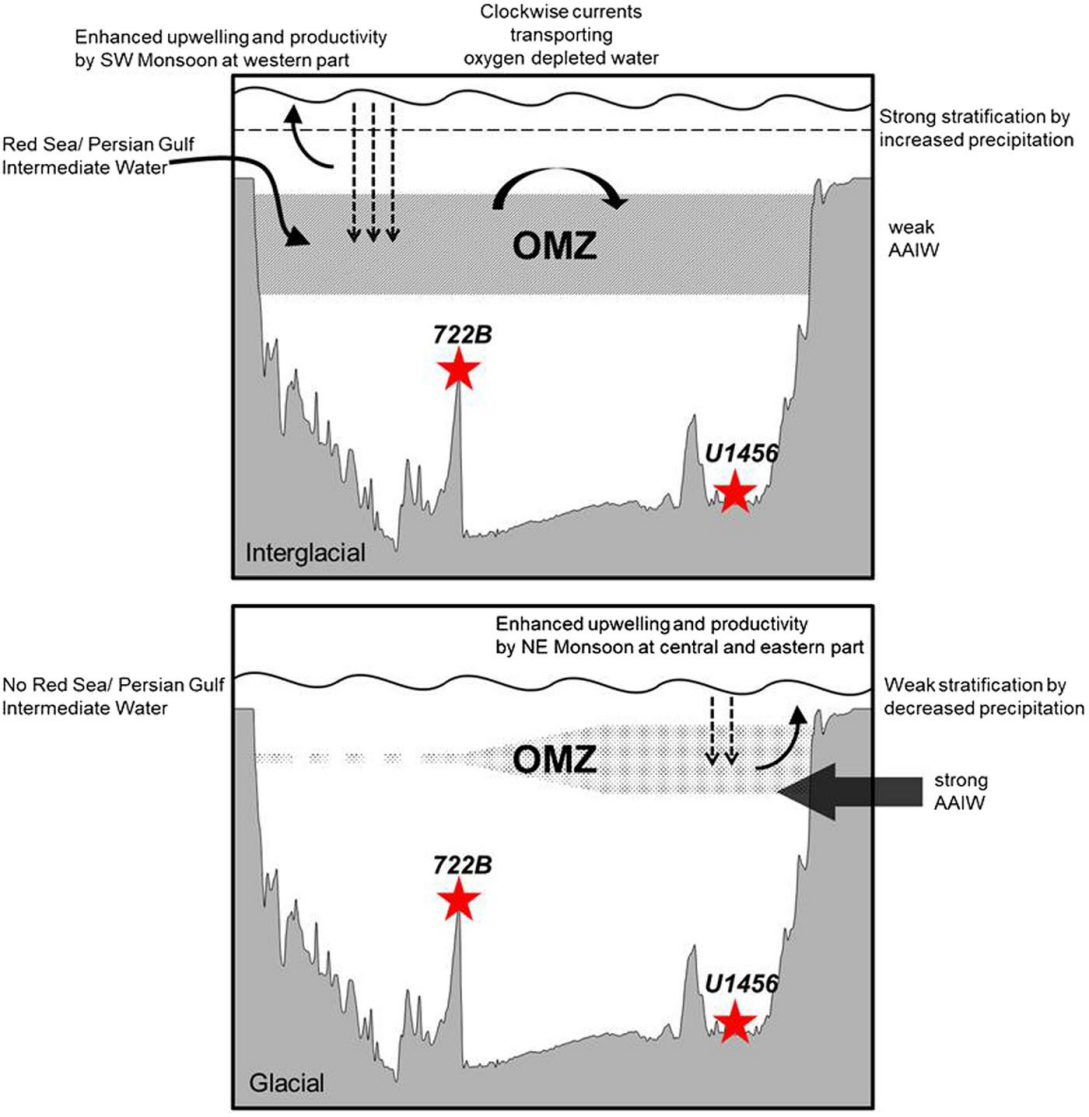
Model of interglacial-glacial denitrification change in association with development of the OMZ in the Arabian Sea. The intensity of the OMZ differs between interglacial and glacial periods^10^. A thicker OMZ and intensified denitrification occurred during interglacial periods, and while the opposite occurred during glacial periods. Because the seasonal OMZ during the glacial periods was weak, denitrification collapsed in the Western Arabian Sea. In contrast, the perennial OMZ has maintained strong denitrification in the Eastern Arabian Sea as indicated by δ^15^N values greater than 6‰.

The degree of denitrification in the Arabian Sea is primarily controlled by several oceanographic factors linked with glacial-interglacial cycles^10,20–22,24^. Figure 4 provides a schematic model demonstrating that the degree of denitrification changed between the interglacial and glacial periods in the Arabian Sea. First, the driving force of OMZ development is closely related to the seasonally-reversing monsoon winds. The southwest monsoon during summer causes seasonal upwelling in the Western Arabian Sea. Similarly, during the warm interglacial periods, the degree of denitrification increased in the Western Arabian Sea^10,20–22,24^. However, the prevailing northeast monsoon during the cold glacial periods caused downwelling in the Western Arabian Sea and upwelling in the Eastern Arabian Sea^29^. Thus, the monsoon-derived upwelling in the Eastern Arabian Sea maintained the perennial OMZ, while the seasonal OMZ collapsed in the Western Arabian Sea. Second, the clockwise-flowing Somali Current plays an important role in transporting oxygen-depleted water throughout the Arabian Sea during the summer monsoon^49,50^ (Fig. 1). Thus, the surface current during the interglacial periods promoted wide OMZ for denitrification throughout the basin^50^. However, the role of the Somali Current was reduced during glacial periods due to the northeast monsoon. The third component controlling the degree of denitrification is the supply of oxygen-depleted waters by the RSIW. During interglacial periods, the RSIW influx from the Arabian Peninsula supplied oxygen-depleted waters to the intermediate layer in the Arabian Sea, which elevated the degree of denitification^10,33,34^. However, during glacial periods when the sea level was lower than the sill depth of the straits, the input of the RSIW ceased, resulting in the reduced denitrification. The fourth factor is the low contribution of relatively oxygen-rich AAIW to the Arabian Sea during the interglacial periods as a result of development of the Banda Sea Intermediate Water. However, when the Banda Sea Intermediate Water was weak during the cold glacial periods, denitrification decreased because of the greater influence of the AAIW, which was able to supply the dissolved oxygen^34,51,52^. Finally, salinity stratification due to increased monsoon precipitation during the southwest monsoon limited the oxygen exchange between the subsurface water and the atmosphere, intensifying the degree of denitrification. In contrast, such stratification was weakened when riverine freshwater discharge decreased during the arid winter monsoon^53^.

In conclusion, the OMZ and denitrification have developed actively to a greater extent during interglacial periods, which are mainly characterized by a strong southwest monsoon in the Arabian Sea. The seasonal OMZ in the Western Arabian Sea collapsed due to the diverse environmental factors during the glacial periods. In contrast, perennial OMZ was maintained in the Eastern Arabian Sea, as indicated by most δ^15^N values of bulk sediments at Site U1456 being higher than the 6‰ denitrification threshold during the last 800 kyrs. The denitrification in the Arabian Sea has also changed in response to climatic fluctuations following the 100-ka cyclicity represented by global δ^18^O values and other multi-proxies. Our results emphasize that the Eastern Arabian Sea has experienced persistent denitrification without prominent collapse throughout the Mid-Pleistocene.

## Methods

For the present study, a total of 260 samples were collected from a composite section of Unit I consisting of Holes U1456A and U1456C at Site U1456. The δ^18^O values were measured from planktonic foraminifera (*Globigerinoides ruber*) with a test size between 250 and 355 μm using IsoPrime at Center for Advanced Marine Core Research of Kochi University (Japan). Cleaning of foraminifera tests was conducted according to Barker *et al*.^54^. Oxygen isotope ratios were calibrated to the V-PDB standard using international standard NBS19. The analytical precision of the δ^18^O values is ±0.06‰. The δ^15^N values of 173 bulk sediments from Unit I were measured using EA-IRMS at Iso-Analytical Ltd. (UK). All δ^15^N values were calibrated to δ^15^N_air_ and the precision was about ±0.1‰. The total nitrogen (TN) content of bulk sediments was measured using CHN Elemental Analyzer (Flash 2000 Model) at Pusan National University (Korea). The analytical precision of TN was ±0.1%. All analytical data are summarized in Supplement Data File.

## Electronic supplementary material

Supplemenatary Figures

Dataset 1
