# Supplementary material for: Orbital-scale denitrification changes in the Eastern Arabian Sea during the last 800 kyrs
Source: Sci Rep. 2018 May 4;8:7027. doi: 10.1038/s41598-018-25415-7 (PMC5935671; doi:10.1038/s41598-018-25415-7)
Supplement: Supplementary file 1 — Supplemenatary Figures [file 41598_2018_25415_MOESM1_ESM.docx]

**Supplementary Figures**

**Orbital-scale denitrification changes in the Eastern Arabian Sea during the last 800 kyrs**

Ji-Eun Kim, Boo-Keun Khim, Minoru Ikehara and Jongmin Lee


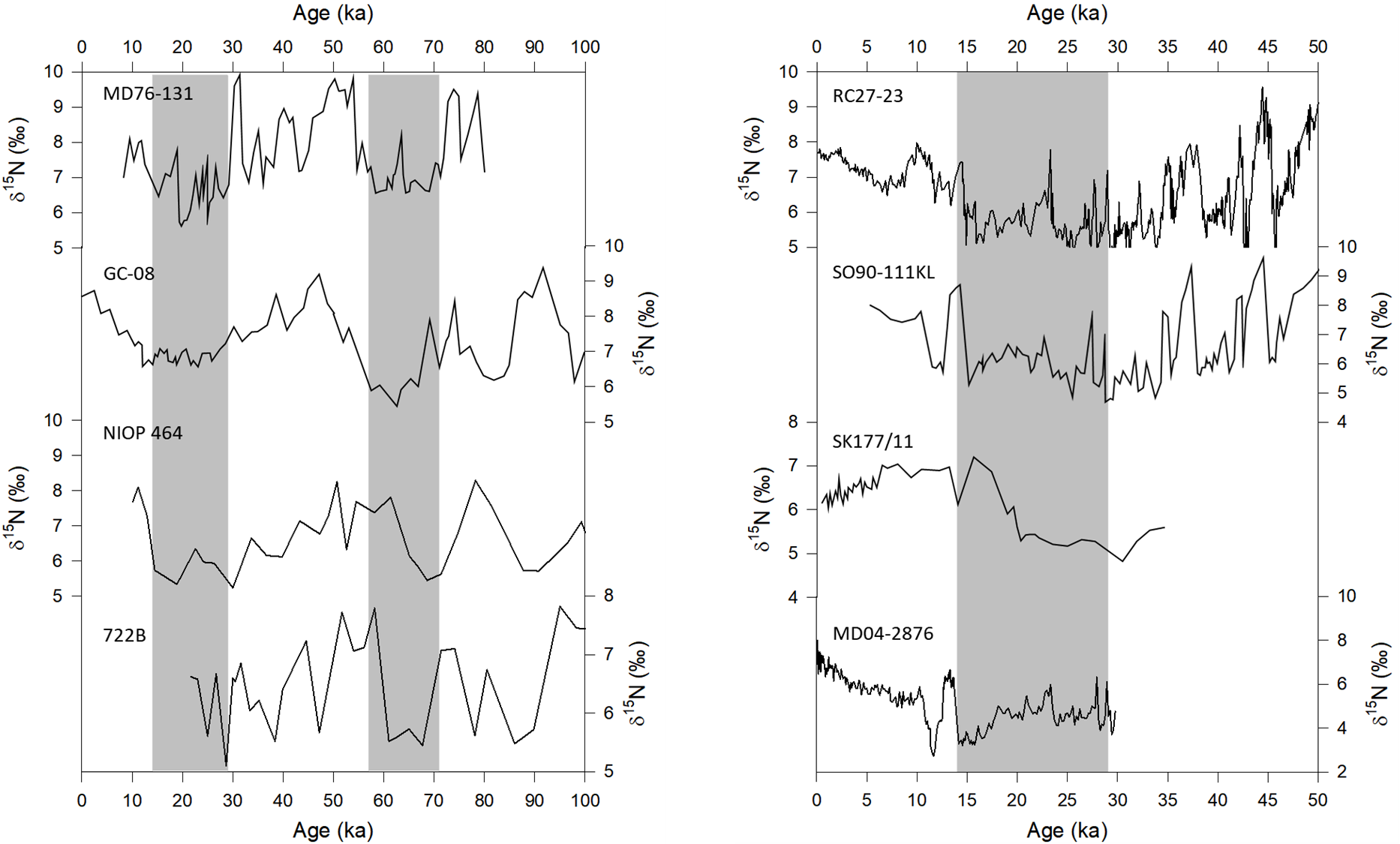


Supplementary Figure S1. Orbital- and millennial-scale denitrification changes in the Arabian Sea^S1-S8^. δ^15^N values of bulk sediments in the Arabian Sea are high during the interglacials and interstadials and low during the glacials and stadials.

**
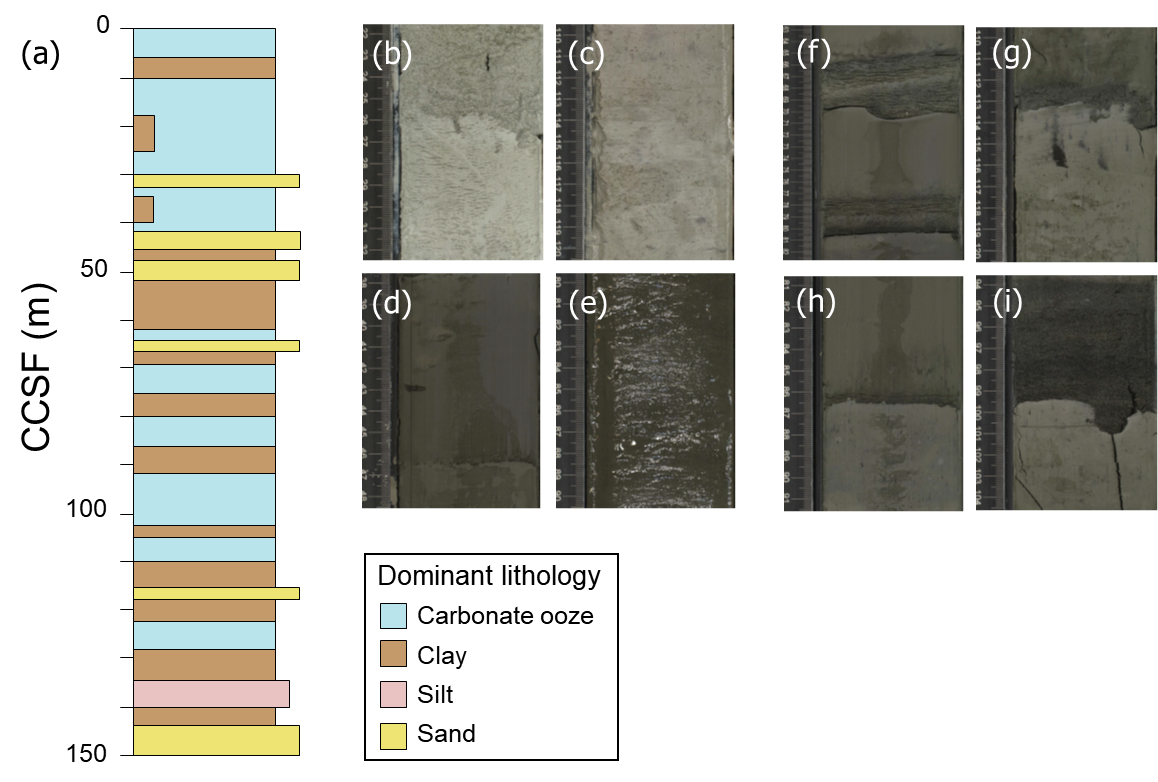
**

Supplementary Figure S2. Lithology of Unit I at Site U1456 with core photographs. (a) Schematic lithologic column of Unit I, mainly consisting of pelagic carbonate ooze and hemipleagic sand, silt and clay. Core photographs show dominant lithologies and sedimentary structures from Hole U1456A such as (a) calcareous ooze (12H-1, 22-32 cm), (c) nannofossil ooze (5H-1, 110-120 cm), (d) nannofossil-rich clay (4H-7, 38-48 cm), (e) sand (4H-4, 80-90 cm). (f) turbidites with normal grading (7H-5, 63-82 cm), (g) oxidized pyrites within nannofossil ooze (10H-1, 110-120 cm), (h) sharp erosional boundary above the nannofossil ooze (7H-4, 81-91 cm), and (i) scoured surfaces by erosion on the nannofossil ooze (10-H, 94-101 cm). All photographs are adopted from Pandey *et al.* ^S9^

Supplementary Figure S3: Determination of tie points based on oxygen and nitrogen isotope stratigraphy

**
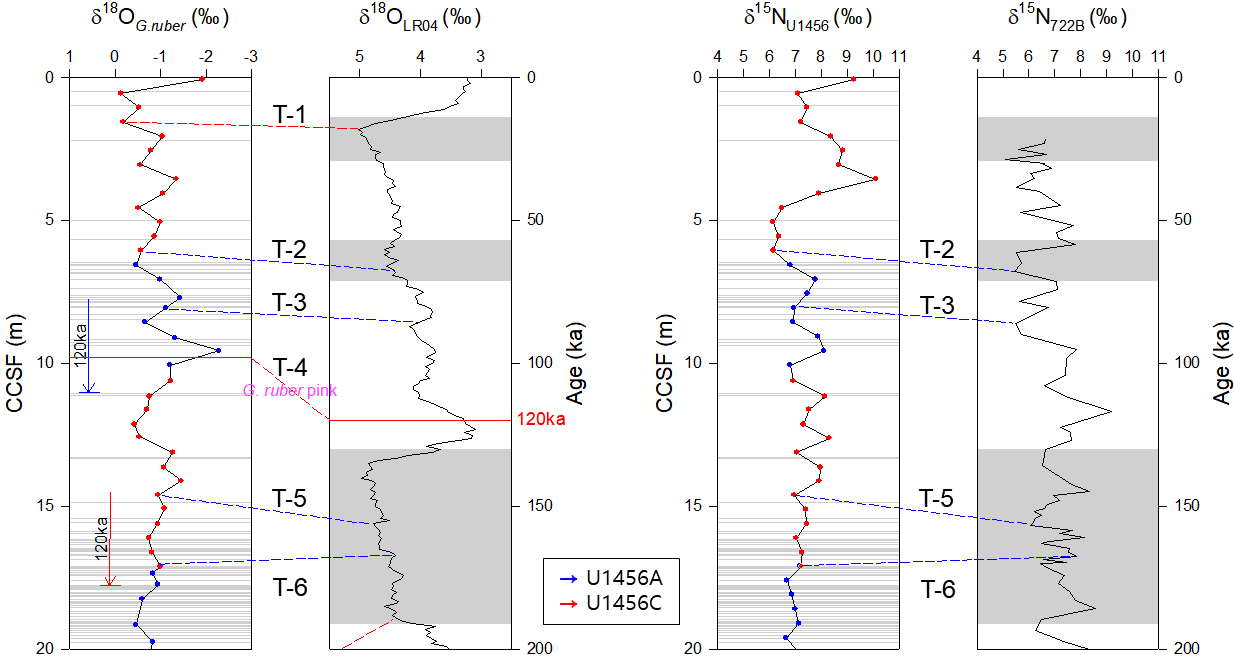
**

Figure S3-1 (0-200 ka). T-1 was determined using the overall sedimentation rate (~14 cm/ka) for correlation of the highest δ^18^O value possibly representing the last glacial maximum. However, the correct age should require AMS ^14^C age later. T-2 correlates the lowest δ^15^N values between Sites U1456 and 722B, belonging to MIS 4, in addition to the consideration of the sedimentation rate. T-3 also correlates the δ^15^N values between Sites U1456 and 722B, based on T-2 and sedimentation rate. T-4 represents the last appearance of pink *Globigerinoides ruber*, indicating 120 ka. It lies within the range of possible last occurrence suggested by the shipboard biostratigrahic data. T-5 was correlated based on the low δ^15^N value of Sites U1456 and 722B. T-6 was determined by the high δ^15^N value of Sites U1456 and 722B.

**
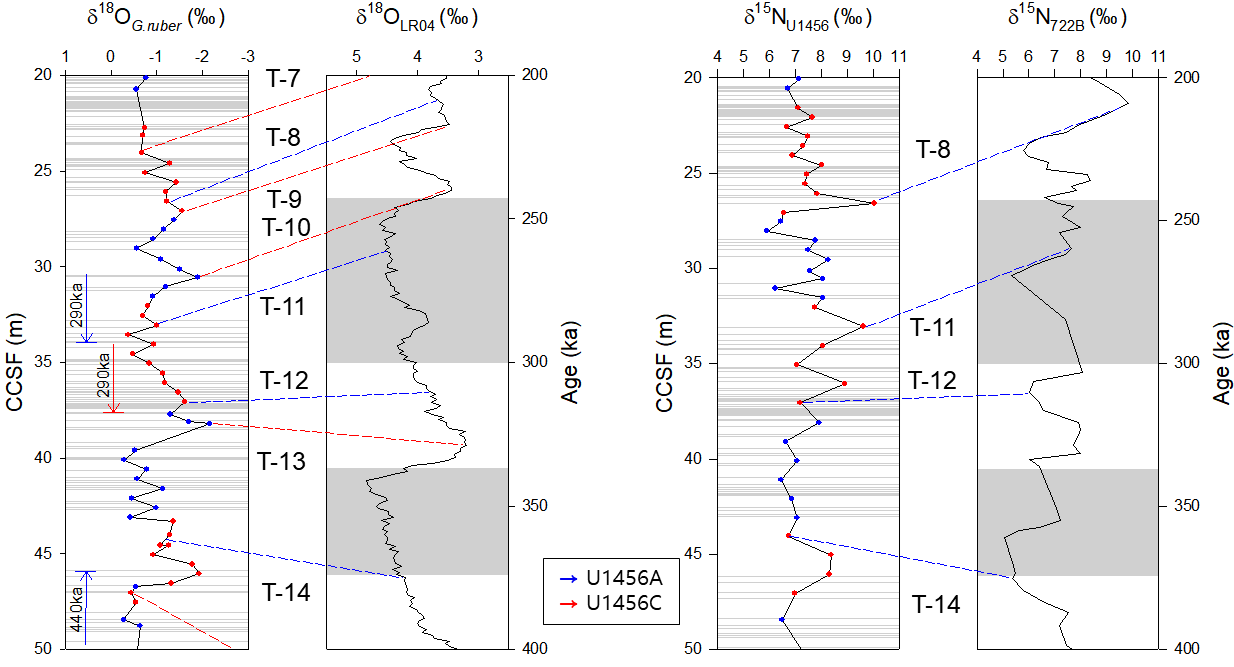
**

Figure S3-2 (200-400 ka). T-7 was correlated by δ^18^O value that changes suddenly between MIS 6 and 7. T-8 was considered by comparing the high interglacial δ^15^N value between Sites U1456 and 722B in MIS 7. T-9 and T-10 were determined by the occurrence of low interglacial δ^18^O value that is observed in both Site U1456 and LR04. Also, the shipboard biostratigraphy data indicating the absence of *Pseudoemiliaia lacunosa* within the interval confirm that the age is younger than 290 ka. T-11 is correlated by the high δ^15^N values observable before T-10 which has been connected by δ^18^O values. T-12 was correlated by the low δ^15^N values between both Sites U1456 and 722B, with the possible consideration of age passing 290 ka. T-13 was correlated by the low δ^18^O values between Site U1456 and LR04, with the age certainly passing 290 ka. T-14 was correlated by the δ^15^N values showing transition from the low to high value from MIS 10 to 11.


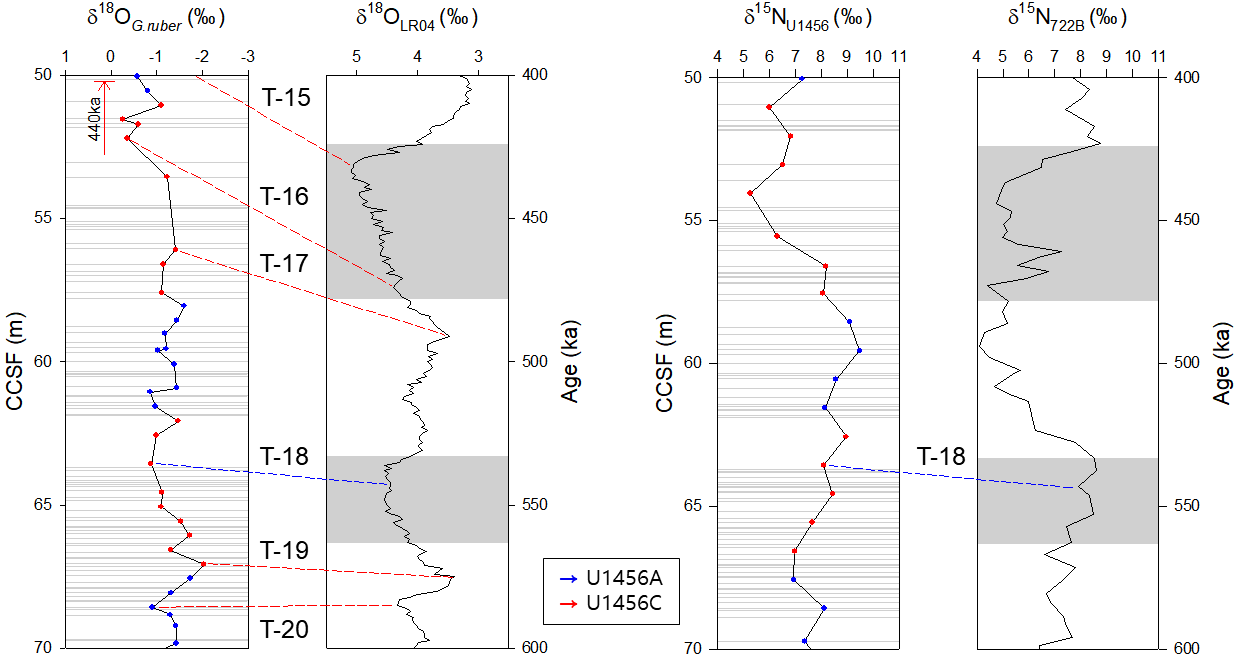


Figure S3-3 (400-600 ka). T-15 was correlated by the high δ^18^O values that mark the transition of MIS 11 and 12. T-16 was determined by δ^18^O values considering the biostratigraphy age range that should be close to 440 ka by the presence of *P. lacunosa*. T-17 is the lowest δ^18^O value at Site U1456 which can be easily correlated to LR04 during MIS 13. T-18 was determined by correlating the low δ^15^N values which occur between two high values in Sites U1456 and 722B. T-19 was matched by the high δ^18^O values which occurred after the low δ^18^O value during MIS 15. T-20 was, on the other hand, correlated by the high δ^18^O value before T-19.


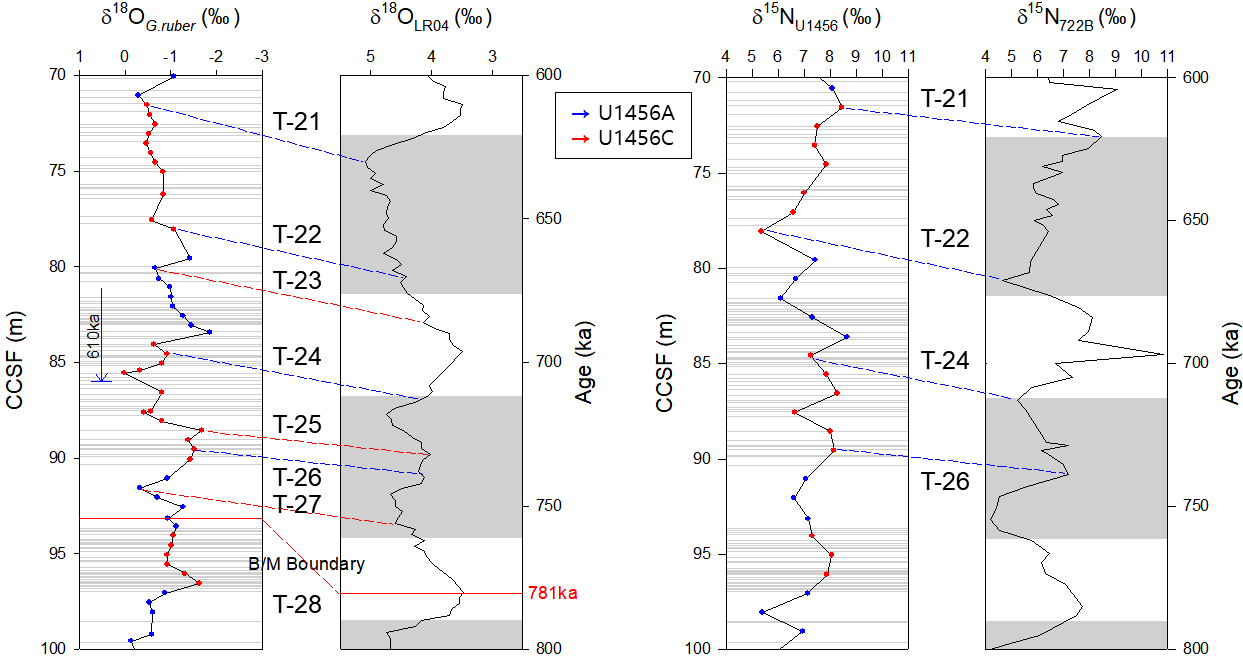


Figure S3-4 (600-800 ka). T-21 was determined by increase of δ^15^N values during MIS 15, based on the sedimentation rate judged by the ages above. T-22 was correlated to the low δ^15^N values which occur before high interglacial δ^15^N value in MIS 16. T-23 was determined by the low δ^18^O values which follow a major δ^18^O peak. T-24 was correlated by the significant low δ^15^N values at both Sites U1456 and 722B. T-25 was correlated for the proceeding δ^18^O peak of the double δ^18^O peaks that occur in LR04. Also, by the last occurrence of *Globorotalia tosaensis*, the age is considered to be older than 610 ka. T-26 was determined mainly by the highest δ^15^N values before T-25 between Sites U1456 and 722B, and the validity can be also checked by δ^18^O values between two sites. T-27 shows corresponding high δ^18^O values that indicate the transition between MIS 18 and 19. T-28 was determined by the shipboard paleomagnetic datum, a horizon that marks the Bruhnes-Matuyama boundary (781 ka) occurring in 93.17 CCSF (m).


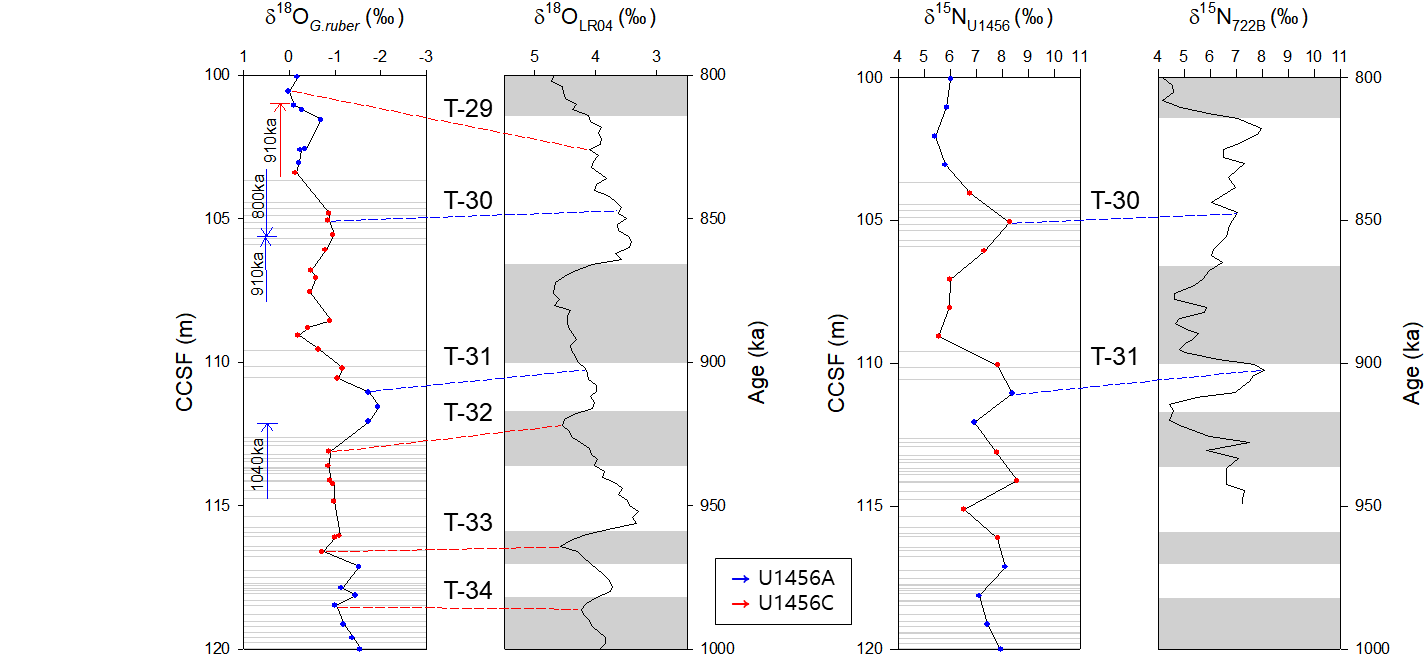


Figure S3-5 (800-1000 ka). T-29 is the high δ^18^O value before the Bruhnes-Matuyama boundary, which is correlated between two data sets. T-30 is the high δ^15^N value of Site U1456, which is correlated to Site 722B that proceeds the decrease of δ^15^N value. Also, the coiling shift in *Pulleniatina* tests indicate that 800 ka should be above T-30. The occurrence of *Reticulofenestra asanoi* supplements 910 ka to appear below T-30. T-31 was determined by the highest δ^15^N values before T-30, with consideration of the proceeding sedimentation rates. Also, the reappearance of *Gephyrocapsa* spp. indicating 1040 ka supports the location of T-31. T-32 was correlated by the high δ^18^O values that shows the transition before T-31. T-33 and T-34 were considered at the current position by comparing the pattern of transition of δ^18^O between Site U1456 and LR04.


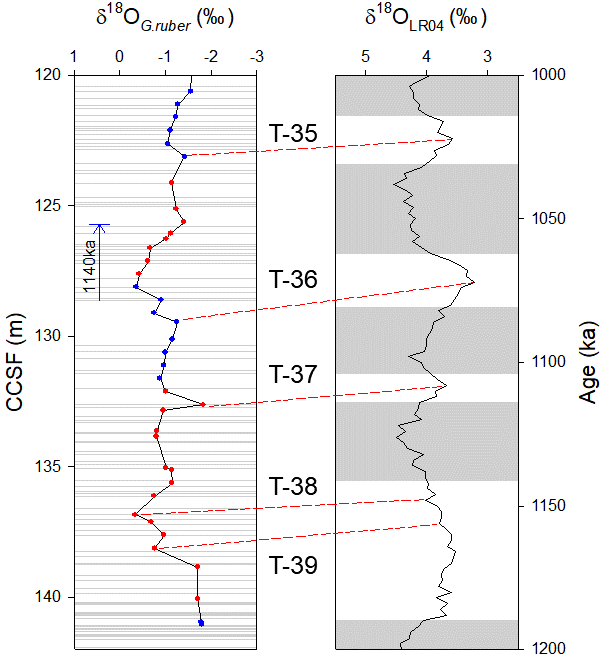


Figure S3-6 (1000-1200 ka). T-35 was correlated by the low δ^18^O values with overall sedimentation rate considered. T-36 and T-37 are the lowest δ^18^O value between Site U1456 and LR04 during MIS 31 and 33, respectively. Also, the ages below are older than 1140 ka by the disappearance of *R. asanoi*. T-38 was assumed by the overall sedimentation rate from the ages above and by the high δ^18^O value between Sites U1456 and 722B during MIS 35. T-39 was correlated by the δ^18^O values with overall sedimentation rate during MIS 36.


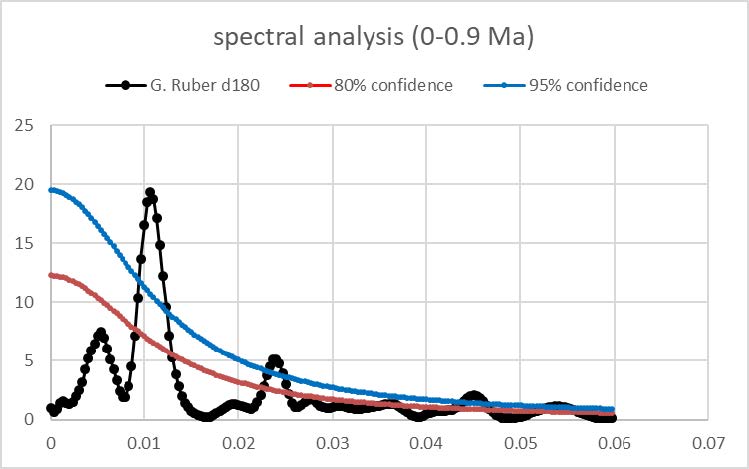


Supplementary Figure S4. Result of spectral analysis for δ^18^O values during the last 0.9 Ma at Site U1456. The most dominant periodicity of δ^18^O values during the last 0.9 Ma at Site U1456 is 100 kyr of eccentricity forcing.


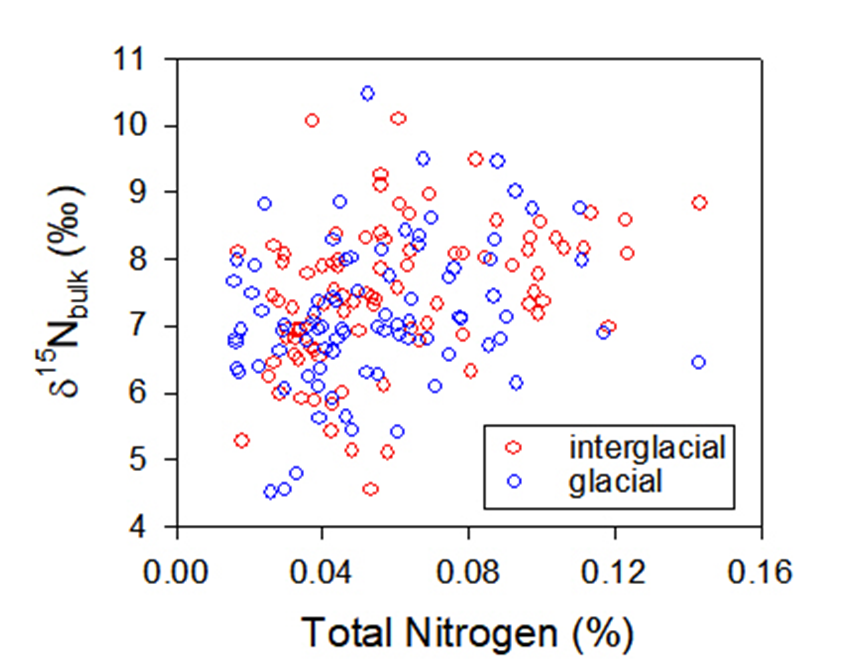


Supplementary Figure S5. The effect of early diagenesis evaluated by comparing δ^15^N value with total nitrogen (TN) content of Unit I at Site U1456. When sedimentary organic matter is degraded within the sediments in an oxic condition, the TN content decreases and δ^15^N value increases, respectively. However, correlation between two parameters of Unit I at Site U1456 is insignificant. Thus, the effect of early diagenesis at this site seems negligible.

S1 Ganeshram, R. S., Pedersen, T. F., Calvert, S. E., McNeill, G. W. & Fontugne, M. R. Glacial‐interglacial variability in denitrification in the World's Oceans: Causes and consequences. *Paleoceanography* **15**, 361-376 (2000).

S3 Banakar, V. *et al.* Monsoon related changes in sea surface productivity and water column denitrification in the Eastern Arabian Sea during the last glacial cycle. *Marine Geology* **219**, 99-108 (2005).

S4 Reichart, G.-J., Lourens, L. & Zachariasse, W. Temporal variability in the northern Arabian Sea Oxygen Minimum Zone (OMZ) during the last 225,000 years. *Paleoceanography* **13**, 607-621 (1998).

S5 Altabet, M. A., Murray, D. W. & Prell, W. L. Climatically linked oscillations in Arabian Sea denitrification over the past 1 my: Implications for the marine N cycle. *Paleoceanography* **14**, 732-743 (1999).

S6 Altabet, M. A., Higginson, M. J. & Murray, D. W. The effect of millennial-scale changes in Arabian Sea denitrification on atmospheric CO_2_. *Nature* **415**, 159-162 (2002).

S7 Suthhof, A., Ittekkot, V. & Gaye‐Haake, B. Millennial‐scale oscillation of denitrification intensity in the Arabian Sea during the Late Quaternary and its potential influence on atmospheric N_2_O and global climate. *Global Biogeochemical Cycles* **15**, 637-649 (2001).

S8 Kao, S.-J. *et al.* Spatiotemporal variations of nitrogen isotopic records in the Arabian Sea. *Biogeosciences* **12**, 1-14 (2015).

S9 Pandey, D. K., Clift, P. D., Kulhanek, D. K. & the Expedition 355 Scientists. Site U1456. In *The Proceedings of the International Ocean Discovery Program* **355**, 1-61, doi:10.14379/iodp.proc.355.103.2016 (2016).
